# Prevalence of burnout and related factors in nursing faculty members: a systematic review

**DOI:** 10.3352/jeehp.2022.19.16

**Published:** 2022-07-14

**Authors:** Marziyeh Hosseini, Mitra Soltanian, Camellia Torabizadeh, Zahra Hadian Shirazi

**Affiliations:** 1 Student Research Committee, School of Nursing and Midwifery, Shiraz University of Medical Sciences, Shiraz, Iran; 2 Community Based Psychiatric Care Research Center, Department of Nursing, School of Nursing and Midwifery, Shiraz University of Medical Sciences, Shiraz, Iran; Hallym University, Korea

**Keywords:** Job satisfaction, Nursing faculty, Prevalence, Professional burnout, Psychosocial intervention

## Abstract

**Purpose:**

The current study aimed to identify the prevalence of burnout and related factors in nursing faculty members through a systematic review of the literature.

**Methods:**

A comprehensive search of electronic databases, including Scopus, PubMed, Web of Science, Iranmedex, and Scientific Information Database was conducted via keywords extracted from Medical Subject Headings, including burnout and nursing faculty, for studies published from database inception to April 1, 2022. The quality of the included studies in this review was assessed using the appraisal tool for cross-sectional studies.

**Results:**

A total of 2,551 nursing faculty members were enrolled in 11 studies. The mean score of burnout in nursing faculty members based on the Maslach Burnout Inventory (MBI) was 59.28 out of 132. The burnout score in this study was presented in 3 MBI subscales: emotional exhaustion, 21.24 (standard deviation [SD]=9.70) out of 54; depersonalization, 5.88 (SD=4.20) out of 30; and personal accomplishment, 32.16 (SD=6.45) out of 48. Several factors had significant relationships with burnout in nursing faculty members, including gender, level of education, hours of work, number of classroom, students taught, full-time work, job pressure, perceived stress, subjective well-being, marital status, job satisfaction, work setting satisfaction, workplace empowerment, collegial support, management style, fulfillment of self-expectation, communication style, humor, and academic position.

**Conclusion:**

Overall, the mean burnout scores in nursing faculty members were moderate. Therefore, health policymakers and managers can reduce the likelihood of burnout in nursing faculty members by using psychosocial interventions and support.

## Introduction

### Background/rationale

Occupations play an important role in people’s lives by providing structure, purpose, and meaning. Also, through their occupations, people can make a difference and have a positive impact on themselves and others [1]. Teaching is one of the professions requiring long periods of work to achieve constant success [2]. Faculty members are in daily contact with students and also work in the field of research [3,4]. In addition to the responsibilities associated with educating nursing students, nursing professors and educators, such as nurses, experience work environment stressors due to their presence in clinical settings [5]. Therefore, successfully balancing areas of activity can be challenging for faculty members. Being active in such a work environment, with too many tasks and too little time, reduces job satisfaction and increases stress [3]. Severe and long-term job stress causes occupational burnout [6].

Burnout is a psychological syndrome that originates from overload and chronic interpersonal stressors caused by the work environment [7]. According to a literature review, burnout includes 3 dimensions: emotional exhaustion (EE), depersonalization (DP), and reduced personal accomplishment (PA) [8]. The results of the study by Xu et al. [9] showed that approximately 85% of nursing faculty members had moderate and high levels of burnout. According to the evidence, burnout syndrome is more common in professionals who work directly with people [10]. Professors’ burnout can have devastating personal and occupational consequences such as low job satisfaction, a low level of commitment, and a tendency to leave the teaching position, which reduces the quality of education [11,12]. Nurses are the largest group of health professionals [13]; therefore, their education is very important.

### Objectives

This study aimed to find the prevalence of burnout and related factors in nursing faculty members through a systematic review of the literature.

## Methods

### Ethics statement

This was not a study on human or human-originated materials; therefore, neither approval by the institutional review board nor the obtainment of informed consent was required.

### Protocol & registration

The present systematic review was conducted based on the Preferred Reporting Items for Systematic Reviews and Meta-Analysis (PRISMA) guidelines [14]. This review study was not registered in the International Prospective Register of Systematic Reviews (PROSPERO) database because its website was undergoing maintenance when we did this research.

### Information sources & search strategy

Electronic databases were searched, including Scopus, PubMed, Web of Science, Iranmedex, and Scientific Information Database with the following search on April 1, 2022:

#1 Burnout: (“Burnout”) OR (“Professional burnout”) OR (“Job burnout”) OR (“Occupational burnout”)
#2 Nursing faculty: (“Faculty”) OR (“Nursing faculty”) OR (“Nursing teachers”) OR (“Nursing educators”) OR (“University professor”) OR (“Nursing professor”)
#3 Combination: #1 AND #2

Persian electronic databases were searched using the equivalent Persian keywords. All search steps were performed by 2 researchers independently. The gray literature, such as conference presentations, expert opinions, dissertations, research and committee reports, and ongoing research, was not included in this systematic review. The term “gray literature” refers to articles produced in print and electronic formats but not reviewed by academic or commercial publishers [15].

### Eligibility criteria

Cross-sectional studies published in English and Persian on burnout and related factors in nursing faculty members were included in this systematic review. Letters to the editor, case reports, conference proceedings, experiments, studies with qualitative designs, and reviews were excluded.

### Study selection

The data of this systematic review were managed using EndNote X8 software (Clarivate, Philadelphia, PA, USA). Research selection criteria, including the elimination of duplicate studies, evaluation of the titles and abstracts of the study, and the full text of the electronic articles, were evaluated manually by 2 researchers (M.H. and C.T.) independently based on the inclusion and exclusion criteria. Any disagreements in the evaluation of the studies between 2 researchers (M.H. and C.T.) were resolved by a third researcher (M.S.). Finally, to prevent data loss, the list of study references was evaluated manually.

### Data collection process, data items, and synthesis of results

The information extracted in this review by the researchers included the name of the first author, the year of publication, location, sample size, male/female ratio, age, single/married ratio, level of education, academic rank, teaching experience, questionnaire, key results, and burnout rate.

### Risk of bias in individual studies & risk of bias across studies

The appraisal tool for cross-sectional studies (AXIS tool) evaluates the quality of the included studies via 20 items with a 2-point Likert scale, including yes (score of 1) and no (score of 0). This tool assesses report quality (7 items), study design quality (7 items), and the possible introduction of biases (6 items). Finally, AXIS rates the quality of studies at 3 levels: high (70% to 100%), fair (60% to 69.9%), and low (0% to 59.9%) [16]. Data extraction and qualitative evaluation of studies were performed by 2 researchers (M.H. and Z.H.S.) independently.

### Summary measures

None.

### Additional analyses

Not available.

## Results

### Study selection

**Fig. 1.**
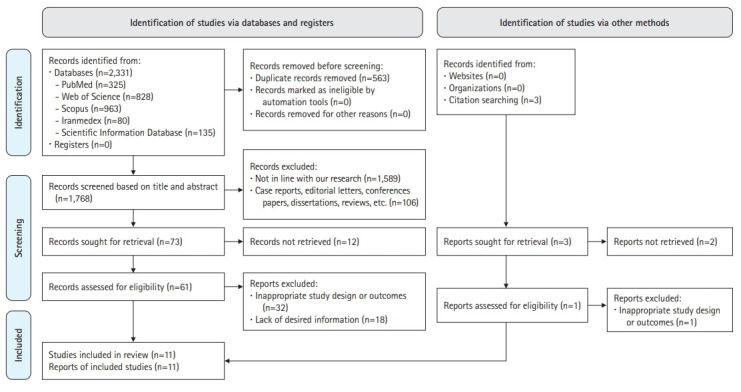
Flow diagram of the study selection process.

As shown in Fig. 1, 2,331 studies were obtained by searching databases. After deleting 563 duplicate studies, 1,768 remained. Due to inconsistency with the purpose of the present review study, 1,589 studies were deleted after reviewing the title and abstract of the articles. Furthermore, 106 studies were excluded due to a non-cross-sectional design. Sixty-one studies were eligible for an evaluation of the full text of the articles. Thirty-two studies were removed due to an inappropriate design or results, and 18 studies were excluded due to a lack of sufficient information after reviewing the full text of the articles. Finally, 11 studies [3-5,9,17-23] remained in this systematic review.

### Study characteristics & results of individual studies

A total of 2,551 nursing faculty members were enrolled in 11 studies [3-5,9,17-23]. The overwhelming majority (89.97%) of nursing educators were women, and 68.78% of them were married. Their mean±standard deviation [SD] age was 41.94±8.49 years. Five studies reported participants’ academic rank, of which 15.50% of nursing faculty members were professors and 15.63% were assistant professors [4,5,18,19,21,23]. Most studies (n=9) used the Maslach Burnout Inventory (MBI) to examine burnout in nursing faculty members [3,4,9,18-23]. The characteristics of the studies are presented in Supplement 1.

### Risk of bias within studies & risk of bias across studies

Of the 11 studies included in this review [3-5,9,17-23], 10 had high-quality studies [3-5,9,17,18,20-23] and 1 study had fair quality [19]. Four studies did not report the selection process [4,17-19], 3 studies did not report research limitations [4,18,19], and 7 studies did not report funding sources or conflicts of interest [4,17-21,23] (Table 1).

### Synthesis of results

#### Burnout in nursing faculty members

The mean score of burnout in nursing faculty members based on the MBI was 59.28 out of 132 [3,4,9,18-23]. The mean±SD scores of the MBI subscales were as follows: EE, 21.24±9.70 out of 54; DP, 5.88±4.20 out of 30; and PA, 32.16±6.45 out of 48.

#### Factors associated with the EE subscale

The factors significantly associated with the EE subscale in the MBI were gender (n=1) [4] and level of education (n=1) [3]. Factors such as hours of work (n=2) [4,22], number of classroom students taught (n=1) [22], full-time work (n=1) [4], job pressure (n=1) [18], perceived stress (n=1) [9], subjective well-being (n=1) [9], and marital status (n=1) [18] had significant positive relationships with the EE subscale in the MBI. However, the EE subscale in the MBI had significant negative relationships with job satisfaction (n=2) [18,22], work setting satisfaction (n=1) [18], workplace empowerment (n=1) [22], collegial support (n=1) [19], management style (n=1) [19], and fulfillment of self-expectations (n=1) [18] (Table 2).

#### Factors associated with the DP subscale

Factor such as marital status (n=1) [18], job pressure (n=1) [18], perceived stress (n=1) [9], subjective well-being (n=1) [9], number of classroom students taught (n=1) [22] had significant positive relationships with the DP subscale in the MBI. However, the DP subscale in the MBI had significant negative relationships with job satisfaction (n=1) [22], workplace empowerment (n=1) [22], communication style (n=1) [18], and management style (n=1) [19] (Table 2).

#### Factors associated with the PA subscale

Factor such as job satisfaction (n=2) [18,22], work setting satisfaction (n=1) [18], workplace empowerment (n=1) [22], management style (n=1) [19], and humor (n=1) [23] had significant positive relationships with the PA subscale in the MBI. However, the PA subscale in the MBI had significant negative relationships with academic position (n=1) [18], perceived stress (n=1) [9], and subjective well-being (n=1) [9] (Table 2).

### Additional analyses

Not available.

## Discussion

### Key results

This systematic review was conducted to summarize burnout and related factors in nursing faculty members. The mean±SD burnout score in this study was presented in three MBI subscales, as follows: that of EE was 21.24±9.70 out of 54, that of DP was 5.88±4.20 out of 30, and that of PA was 32.16±6.45 out of 48. Overall, the mean scores of burnout in nursing faculty members were moderate. Factors such as gender, level of education, hours of work, number of classroom students taught, full-time work, job pressure, perceived stress, subjective well-being, marital status, job satisfaction, work setting satisfaction, workplace empowerment, collegial support, management style, fulfillment of self-expectation, communication style, humor, and academic position had significant relationships with burnout in nursing faculty members.

The results of the present systematic review showed that burnout in nursing faculty members was at a moderate level. However, differences in their level of burnout might have been due to factors such as gender, level of education, hours of work, number of classroom students taught, full-time work, job pressure, perceived stress, subjective well-being, marital status, job satisfaction, work setting satisfaction, workplace empowerment, collegial support, management style, fulfillment of self-expectations, communication style, humor, and academic position.

### Comparison with previous studies

A study in Iran showed that faculty members at a medical school had high levels of DP and EE. Furthermore, it was found that there were significant positive correlations between DP and executive work experience and between EE severity and gender [24]. Another study reported in South Korea that approximately one-third of faculty members had high level of burnout in all sub-dimensions. DP and the EE levels were also significantly higher in women or people younger than 40 years, and long working hours were the factor that most strongly reduced the PA scores.

In that study, excessive regulation by the government or university was introduced as the most substantial cause of burnout [25]. Furthermore, the result of a literature review by Thomas et al. [26] showed that nursing faculty members have needs related to student guidance and counseling. The results of that study showed that excessive work stress and imbalance between work and life can increase the risk of burnout. Burnout was also higher in faculty members with short work experience, and women also experienced more burnout than men [26]. In the study of Seo et al. [27], variables such as weekly working hours, health status, work experience and age were identified as factors that increased burnout. However, a study of dental faculty members showed that there was no significant relationship between work experience and working hours per week [28]. Another systematic review of the prevalence of burnout among university professors showed that the rate of burnout increased over time. It also showed that psychological demand among professors had increased in the last decade [10]. A study in Brazil showed that occupational burnout had a negative effect on faculty members’ quality of life and, consequently, could affect the quality of the education provided [29]. Therefore, according to the results of studies, the implementation of educational interventions and psychosocial support to reduce the rate of burnout among nursing professors is essential.

### Limitations

Although this systematic review was conducted based on the PRISMA checklist, it was not registered in the PROSPERO database, and a public protocol does not exist because its website was under maintenance when we conducted this research. Due to the methodological diversity and the existence of different tools in the studies, it was not possible to perform a meta-analysis in this systematic review. The lack of a meta-analysis may reduce the accuracy of data analysis and increase the heterogeneity of findings. Although a meta-analysis was not performed in this review, the systematic approach to data collection, sorting, and analysis of studies was a strength. Despite a comprehensive search of databases, all studies in this area may not have been found. Finally, in this systematic review, only studies in English and Persian were included and articles in other languages may not have been included in this study.

### Implications for nursing managers and policymakers

Burnout is an important issue among nursing faculty members because it can reduce their quality of work and cause psychosocial problems. Health policymakers and managers can reduce the likelihood of burnout in nursing faculty members by using psychosocial interventions and support.

### Implications for future research

Based on the results of the present systematic review, it is suggested that future studies use educational and supportive solutions to reduce the risk of burnout in nursing faculty members by focusing on the factors associated with burnout.

### Conclusion

According to the above results, the level of burnout among nursing faculty members was moderate. However, differences in their level of burnout might occur due to factors such as gender, level of education, hours of work, number of classroom students taught, full-time work, job pressure, perceived stress, subjective well-being, marital status, job satisfaction, work setting satisfaction, workplace empowerment, collegial support, management style, fulfillment of self-expectation, communication style, humor, and academic position. Therefore, nursing policymakers should pay special attention to these factors related to the use of nursing faculty members’ health maintenance and promotion programs to increase the quality of nursing education for nursing students. Furthermore, health policymakers and managers can reduce the likelihood of burnout in nursing faculty members by using psychosocial interventions and support.

## Figures and Tables

**Table 1.** Assessment of the quality of the included articles

Section	Assessment item	Dick [19] (2012)	Talbot [23] (2000)	Çam [18] (2001)	Sarmiento et al. [22] (2004)	Kizilci et al. [4] (2012)	Heydari et al. [20] (2014)	Mohammad et al. [21] (2014)	Batista et al. [17] (2016)	Aquino et al. [3] (2018)	Wu et al. [5] (2021)	Xu et al. [9] (2021)
Introduction	Clear aims	+	+	+	+	+	+	+	+	+	+	+
	Appropriate design	+	+	+	+	+	+	+	+	+	+	+
Methods	Sample size justified	+	+	+	+	+	+	+	+	+	+	+
	Population defined	+	+	+	+	+	+	+	+	+	+	+
	Sample representative of population	+	+	+	+	+	+	+	+	+	+	+
	Selection process representative	-	+	-	+	-	+	+	-	+	+	+
	Measures to address non-responders	-	-	-	-	-	-	-	-	-	-	-
	Appropriate outcome variables	+	+	+	+	+	+	+	+	+	+	+
	Valid measures	+	+	+	+	+	+	+	+	+	+	+
	Defined statistical significance	+	+	+	+	+	+	+	+	+	+	+
	Methods described	+	+	+	+	+	+	+	+	+	+	+
Results	Results data described	+	+	+	+	+	+	+	+	+	+	+
	Concerns about non-response bias	-	-	-	-	-	-	-	-	-	-	-
	Non-responder information described	-	-	-	-	-	-	-	-	-	-	-
	Results internally consistent	+	+	+	+	+	+	+	+	+	+	+
	Results presented for analyses	+	+	+	+	+	+	+	+	+	+	+
Discussion	Conclusions justified	+	+	+	+	+	+	+	+	+	+	+
	Limitations identified	-	+	-	+	-	+	+	+	+	+	+
Others	Funding sources or conflicts of interests	-	-	-	+	-	-	-	-	+	+	+
	Ethical approval/consent obtained	-	-	+	+	+	-	+	+	+	+	+

+: Presence of the assessment item.

**Table 2.** Factors associated with burnout among nursing educators

First author/year	Factors associated with the EE subscale	Factors associated with the DP subscale	Factors associated with the PA subscale
Dick [19] (1992)	- There was a significant negative relationship between management style and the EE subscale (r=-0.34, P<0.001).	- There was a significant negative relationship between management style and the DP subscale (r=-0.27, P<0.001).	- There was a significant positive relationship between management style and the PA subscale (r=0.16, P<0.01).
	- There was a significant negative relationship between collegial support and the EE subscale (r=-0.43, P<0.001).	- There was a significant negative relationship between collegial support and the DP subscale (r=-0.26, P<0.001).	
Talbot [23] (2000)	NA	NA	- There was a positive relationship between humor and the PA subscale (r=0.36, P<0.002).
Cam [18] (2001)	- There was a significant negative relationship between work setting satisfaction and the EE subscale (β=0.343, P<0.01).	- There was a significant positive relationship between job pressure and the DP subscale (β=-0.269, P<0.01).	- There was a significant positive relationship between job satisfaction and the PA subscale (β=-0.232, P<0.01).
	- There was a significant negative relationship between job satisfaction and the EE subscale (β=0.296, P<0.01).	- There was a significant negative relationship between communication style and the DP subscale (β=0.246, P<0.01).	- There was a significant negative relationship between academic position and the PA subscale (β=0.266, P<0.01).
	- There was a significant positive relationship between job pressure and the EE subscale (β=-0.207, P<0.01).	- There was a significant positive relationship between marital status and the DP subscale (β=-0.171, P<0.01).	- There was a significant positive relationship between work-setting satisfaction and the PA subscale (β=-0.255, P<0.01).
	- There was a significant positive relationship between marital status and the EE subscale (β=-0.177, P<0.01).		
	- There was a significant negative relationship between the fulfillment of self-expectations and the EE subscale (β=0.161, P<0.01).		
Sarmiento et al. [22] (2004)	- There was a significant negative relationship between workplace empowerment and the EE subscale (r=-0.51, P<0.01).	- There was a significant negative relationship between workplace empowerment and the DP subscale (r=-0.40, P<0.01).	- There was a significant positive relationship between workplace empowerment and the PA subscale (r=0.38, P<0.01).
	- There was a significant negative relationship between job satisfaction and the EE subscale (r=-0.65, P=0.01).	- There was a significant negative relationship between job satisfaction and the DP subscale (r=-0.52, P=0.01).	- There was a significant positive relationship between job satisfaction and the PA subscale (r=0.42, P=0.01).
	- There was a significant positive relationship between the number of classroom students taught and the EE subscale (r=0.38, P<0.05).	- There was a significant positive relationship between the number of classroom students taught and the DP subscale (r=0.38, P<0.05).	
	- There was a significant positive relationship between hours of work per week and the EE subscale (r=0.30, P<0.05).		
Kizilci et al. [4] (2012)	NA	- The results showed that single academics had a higher level of DP than married (P<0.05).	- The results showed that academics 30 years and below reported a lower level of PA than 31 and above of academics (P<0.05).
			- The results showed that professors and research assistants reported a lower level of PA than instructors (P<0.05).
Heydari et al. [20] (2014)	- There was a significant relationship between gender and the EE subscale (P<0.001).	- There was a significant negative relationship between the score of the work environment subscales and the DP subscale (P<0.05).	-
	- There was a significant negative relationship between the score of the work environment subscales and the EE subscale (P<0.05).		
	- There was a significant positive relationship between hours of work and the EE subscale (r=0.21, P=0.01).		
	- There was a significant positive relationship between full-time work and the EE subscale (r=0.37, P<0.001).		
Mohammed et al. [21] (2014)	NA	NA	NA
Batista et al. [17] (2016)	NA	NA	NA
Aquino et al. [3] (2018)	The mean score for the EE subscale among PhD faculty was significantly higher than among DNP faculty (t=1.96, df=144, P=0.025)	NA	NA
Wu et al. [5] (2021)	NA	NA	NA
Xu et al. [9] (2021)	- There was a significant positive relationship between perceived stress and the EE subscale (r=0.483, P<0.01).	- There was a significant positive relationship between perceived stress and the DP subscale (r=0.307, P<0.01).	- There was a significant negative relationship between perceived stress and the PA subscale (r=-0.395, P<0.01).
	- There was a significant negative relationship between subjective well-being and the EE subscale (r=-0.339, P<0.01).	- There was a significant negative relationship between subjective well-being and the DP subscale (r=-0.231, P<0.01).	- There was a significant positive relationship between subjective well-being and the PA subscale (r=0.330, P<0.01).

EE, emotional exhaustion; DP, depersonalization; PA, personal accomplishment; NA, not applicable; PhD, Doctor of Philosophy; DNP, Doctor of Nursing Practice; df, degrees of freedom.

## Supplementary materials

Data files are available from Harvard Dataverse: https://doi.org/10.7910/DVN/VYABPE

Supplement 1. Basic characteristics of the included studies in this systematic review.

Supplement 2. Audio recording of the abstract.
