# Supplementary material for: Prevalence of burnout and related factors in nursing faculty members: a systematic review
Source: J Educ Eval Health Prof. 2022 Jul 14;19:16. doi: 10.3352/jeehp.2022.19.16 (PMC9534603; doi:10.3352/jeehp.2022.19.16)
Supplement: Supplementary file 1 — Supplement 1. Basic characteristics of the included studies in this systematic review. [file jeehp-19-16-suppl.docx]

A supplement for the data source

**Table #1.** Search strategy.

|  | Category | Term | Web of Science | PubMed | Scopus | Iranmedex | Scientific Information Database |
| --- | --- | --- | --- | --- | --- | --- | --- |
| #1 | Burnout | (“Burnout”) OR (“Professional burnout”) OR (“Job burnout”) OR (“Occupational burnout”) |  |  |  |  |  |
| #2 | Nursing faculty | (“Faculty”) OR (“Nursing faculty”) OR (“Nursing teachers”) OR (“Nursing educators”) OR (“University professor”) OR (“Nursing professor”) |  |  |  |  |  |
| #3 | Combination | #1 AND #2 | 828 | 325 | 963 | 80 | 135 |

**Table #2.** Details search terms in databases.

|  | Databases | Search strategy |
| --- | --- | --- |
| #1 | Scopus | ((“Burnout”) OR (“Professional burnout”) OR (“Job burnout”) OR (“Occupational burnout”) AND (“Faculty”) OR (“Nursing faculty”) OR (“Nursing teachers”) OR (“Nursing educators”) OR (“University professor”) OR (“Nursing professor”)). |
| #2 | PubMed | ((“Burnout”) OR (“Professional burnout”) OR (“Job burnout”) OR (“Occupational burnout”) AND (“Faculty”) OR (“Nursing faculty”) OR (“Nursing teachers”) OR (“Nursing educators”) OR (“University professor”) OR (“Nursing professor”)). |
| #3 | Web of Science | ((“Burnout”) OR (“Professional burnout”) OR (“Job burnout”) OR (“Occupational burnout”) AND (“Faculty”) OR (“Nursing faculty”) OR (“Nursing teachers”) OR (“Nursing educators”) OR (“University professor”) OR (“Nursing professor”)). |
| #4 | Iranmedex | ((“Burnout”) OR (“Professional burnout”) OR (“Job burnout”) OR (“Occupational burnout”) AND (“Faculty”) OR (“Nursing faculty”) OR (“Nursing teachers”) OR (“Nursing educators”) OR (“University professor”) OR (“Nursing professor”)). |
| #5 | Scientific Information Database | ((“Burnout”) OR (“Professional burnout”) OR (“Job burnout”) OR (“Occupational burnout”) AND (“Faculty”) OR (“Nursing faculty”) OR (“Nursing teachers”) OR (“Nursing educators”) OR (“University professor”) OR (“Nursing professor”)). |
